# Class II malocclusion nonextraction treatment with growth control*

**DOI:** 10.1590/2176-9451.19.6.113-122.bbo

**Published:** 2014

**Authors:** Zilda Lúcia Valentim Assunção

**Affiliations:** 1 State University of Rio de Janeiro, Specialist in Orthodontics and Facial Orthopedics, State University of Rio de Janeiro (UERJ). Certified by the Brazilian Board of Orthodontics and Facial Orthopedics (BBO)

**Keywords:** Angle Class III malocclusion, Palatal expansion technique, Growth and development

## Abstract

The present study reports a case of Angle Class II malocclusion treatment of a male growing patient with 10-mm overjet, excessive overbite and transverse maxillary deficiency. The case was presented to the Brazilian Board of Orthodontics and Dentofacial Orthopedics (BBO), with DI equal to or greater than 10, as a requirement for the title of certified by the BBO.

## Abstract (pt)

O presente relato de caso aborda o tratamento de uma má oclusão de Classe II de Angle, em paciente do sexo masculino, em fase de crescimento, com sobressaliência de 10mm, sobremordida exagerada e deficiência maxilar transversa. Esse caso foi apresentado à Diretoria do Board Brasileiro de Ortodontia e Ortopedia Facial (BBO), representando a categoria com índice de grau de complexidade (IGC) igual ou acima de dez pontos, como parte dos requisitos para obtenção do título de Diplomada pelo BBO.

## INTRODUCTION

The present study reports the case of a male patient referred to orthodontic treatment at the age of 11 years and 8 months old. His chief complaint was being "too toothy". The patient had a nasal breathing pattern, with history of allergy, onychophagia and adenoidectomy at the age of four. He also had good oral hygiene and was monitored by a pediatric dentist every 6 months. He reported having suffered dental trauma at the age of eight, which resulted in minor fracture on the incisal edge of the left maxillary central incisor. 

## DIAGNOSIS

**Figure 1.**
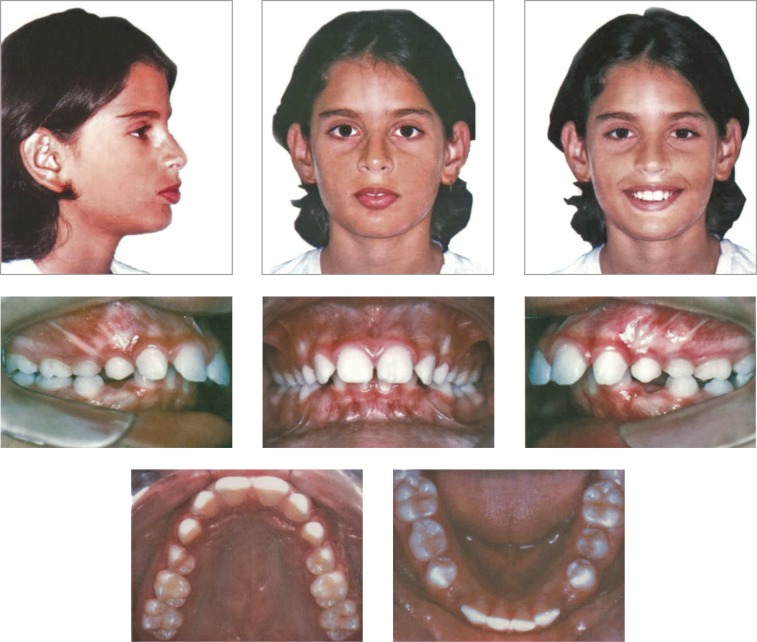
Initial facial and intraoral photographs.

**Figure 2.**
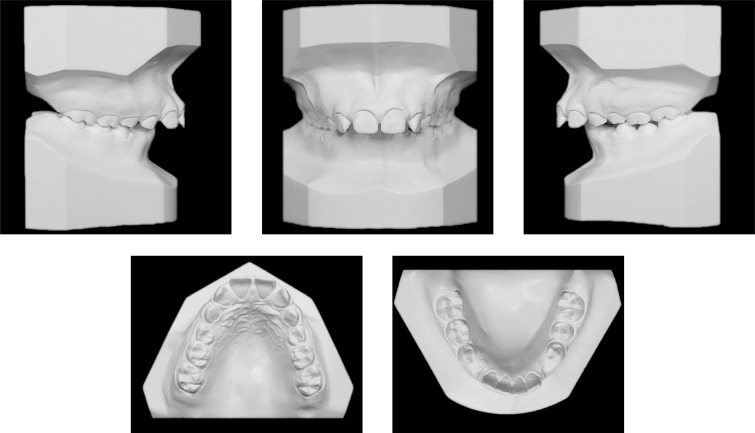
Initial casts.

As shown in Figure 1, facial analysis revealed a convex profile, with a well-defined mentolabial sulcus and everted lower lip (Ul S-line: 1 mm; Ll S-line: 2 mm). Labial seal was strained and, at rest, maxillary incisors were prominent. At smiling, the patient had a wide buccal corridor despite satisfactory smile arc. As shown in Figures 1 and 2, the patient was in mixed dentition, with Class II molar relationship and maxillary incisors bucally tipped, which features Angle Class II, division 1 malocclusion.

He had narrow maxillary arch with high palate; and mandibular arch with accentuated curve of Spee. Maxillary and mandibular midlines were coincident with each other and with the facial midline; severe overbite and overjet were evinced in 10 mm.

**Figure 3.**
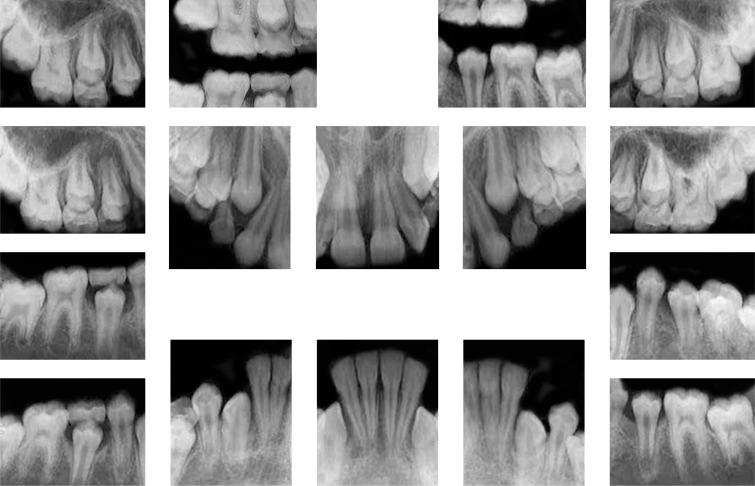
Initial periapical radiographs.

**Figure 4.**
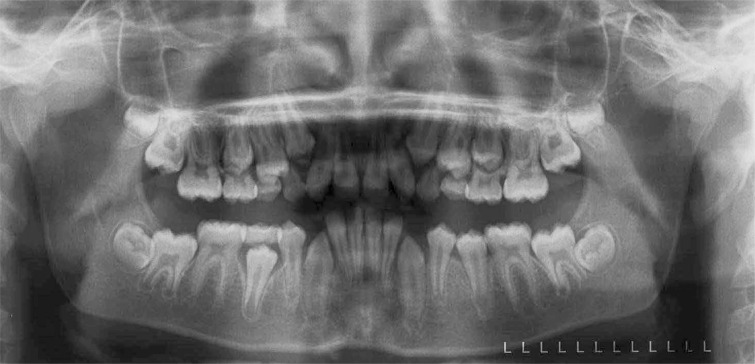
Initial panoramic radiograph.

**Figure 5.**
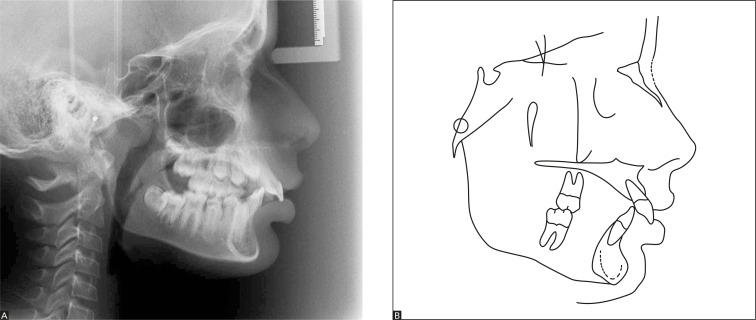
Initial lateral cephalogram (A) and cephalometric tracing (B).

Periapical and panoramic radiographs (Figs 3 and 4) revealed the presence of all permanent teeth, including third molars; in addition to normal tooth as well as bone structures. Cephalometric examination (Fig 5 and Tab 1) revealed that the patient had Class II skeletal pattern (ANB = 6^o)^, protrusion of the maxilla (SNA = 84^o)^ and retrusion of the mandible (SNB = 78^o)^ in relation to the base of the skull. He had decreased mandibular plane with a tendency towards horizontal growth (SN-GoGn = 23^o^ and FMA = 17^o)^. Maxillary incisors were severely buccally tipped and protruded (1-NA = 30^o^ and 8 mm), while mandibular incisors were well positioned (1-NB = 27^o^ and 5 mm).

**Table 1.** Initial (A), intermediate (A1) and final (B) cephalometric values.

	Measurements		Normal	A	B	Dif. A/B
Skeletal pattern	SNA	(Steiner)	82°	84°	81°	3
	SNB	(Steiner)	80°	78°	80°	2
	ANB	(Steiner)	2°	6°	1°	5
	Angle of convexity	(Downs)	0°	8°	-5°	13
	Y axis	(Downs)	59°	63°	59°	4
	Facial angle	(Downs)	87°	87°	88°	1
	SN-GoGn	(Steiner)	32°	23°	23°	0
	FMA	(Tweed)	25°	17°	19°	2
Dental pattern	IMPA	(Tweed)	90°	106°	106°	0
	1.NA (degrees)	(Steiner)	22°	30°	25°	5
	1-NA (mm)	(Steiner)	4 mm	8 mm	5 mm	3
	1.NB (degrees)	(Steiner)	25°	27°	30°	3
	1-NB (mm)	(Steiner)	4 mm	5 mm	3 mm	2
	- Interincisal angle	(Downs)	130°	119°	126°	7
	1-APo	(Ricketts)	1 mm	0 mm	-1 mm	1
Profile	Upper lip — S-line	(Steiner)	0 mm	1 mm	-3 mm	4
	Lower lip — S-line	(Steiner)	0 mm	2 mm	-3 mm	5

**Figure 6.**
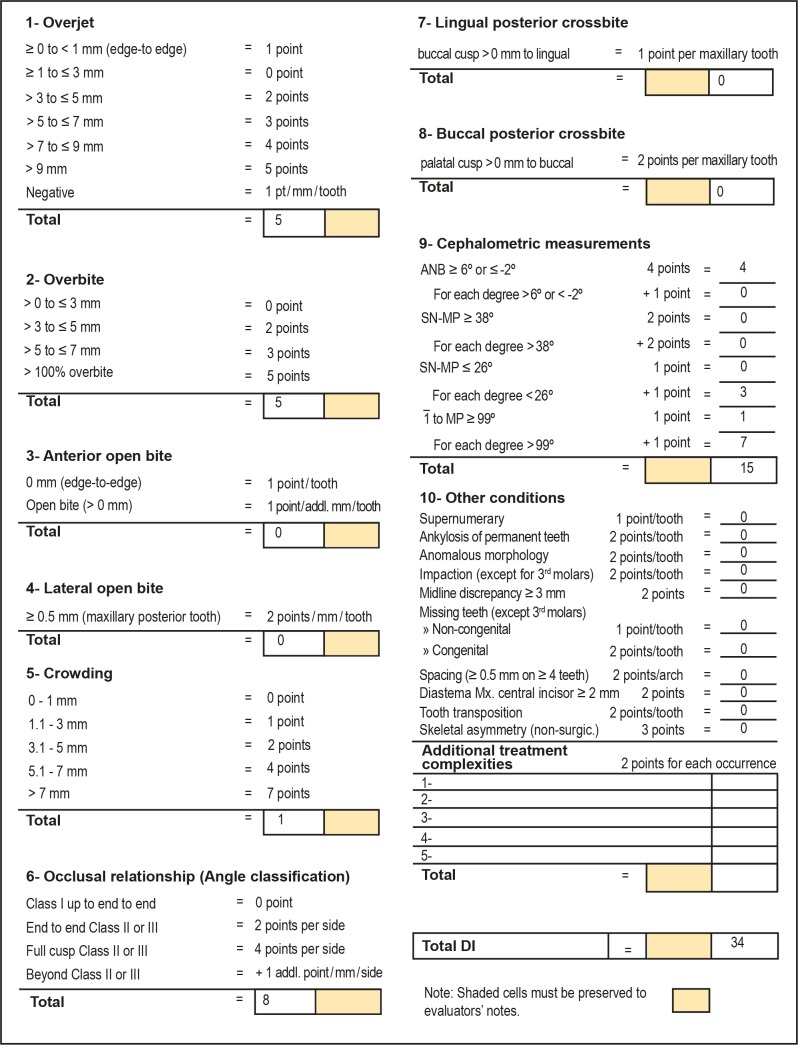
Discrepancy index (DI) calculation.

Discrepancy index (DI) was calculated and scored 34 points (Fig 6).

## TREATMENT PLAN

Before occlusal treatment planning was developed, the patient was referred to an ear, nose and throat specialist for previous evaluation. Despite history of adenoidectomy, patient's lateral cephalogram (Fig 5) revealed adenoid tissue on posterior pharyngeal wall. Thus, there was an urgent need to investigate whether adenoid or other type of tissue was obstructing patient's upper airways and, therefore, causing nasal breathing. Nevertheless, obstruction was absent; thereby rendering nasal breathing an acquired habit associated with hindered labial seal due to severe overjet. Should normal breathing not have been restored after incisor retraction and achievement of potential passive labial seal, the patient would have been referred to speech therapy. Likewise, he would have been referred to psychological therapy to quit nail biting (onychophagia).

With a view to correcting skeletal and dental disharmonies, initial treatment planning consisted of using a Haas appliance to expand the maxilla in transverse direction. Additionally, with a view to correcting skeletal and dental disharmonies in the sagittal plane and to attain Class I molar relationship, treatment planning included the use of Kloehn-type headgear (cervical pull). Thus, treatment would take advantage of patient's favorable growth pattern. Subsequently, to correct severe overbite, an interocclusal splint was installed. A preadjusted, fixed orthodontic Roth prescription appliance with 0.022 x 0.028-in slots was installed for alignment and leveling, incisors retraction and treatment finishing. After the active phase of treatment, a wraparound removable appliance, with anterior bite plate and mandibular intercanine bar, was prescribed.

## TREATMENT PROGRESS

Treatment began by adapting the orthodontic rings used to manufacture the Haas appliance. Subsequently, the appliance was installed and the patient advised to turn the screw 1/4 of a turn every 12 hours during 15 days in order to enhance maxillary shape. Once stability was attained, the expansion appliance remained in position for four months, acting as a retainer. During this period, the Kloehn-type headgear (cervical pull) was installed and used for 16 to 18 hours a day. After removing the expansion appliance, an acrylic plate was installed with a view to aiding severe overbite correction. The patient and his family were informed about the need for compliance, particularly with regard to the headgear and the acrylic plate, necessary to achieve treatment objectives.

During the same phase of treatment, preadjusted brackets (Roth prescription, 0.022 x 0.028-in slots) were bonded to mandibular first molars and incisors for intrusion by means of Ricketts^1^ 0.017 x 0.025-in stainless steel utility arch. After intrusion and with premolars, maxillary canines and second molars erupted; maxillary and mandibular teeth were all bonded. Alignment and leveling were attained by means of stainless steel 0.016, 0.018 and 0.020-in archwire with mild step down bends at the region of mandibular incisors aimed at remaining intruded. Importantly, since treatment onset, both maxillary and mandibular archwires were often used in coordination. In the maxillary arch, retraction of incisors was carried out by means of a stainless steel 0.019 x 0.026-in archwire with a bull loop placed between lateral incisors and canines and used for space closure. After closing existing spaces, the finishing phase was carried out in both arches with the use of stainless steel 0.018 x 0.025-in straight archwires of individual shape, torque and coordination.

After confirming that all treatment objectives had been achieved, both maxillary and mandibular fixed appliances were removed and the retention phase started. To this end, a wraparound removable splint consisting of an stainless steel 0.032-in archwire and an anterior bite plate was installed in the maxillary arch with a view to preventing overbite relapse. As for the mandibular arch, a 0.032-in stainless steel wire intercanine bar was installed. Importantly, the patient proved highly compliant during the active phase of treatment as well as during the retention phase.

## RESULTS

**Figure 7.**
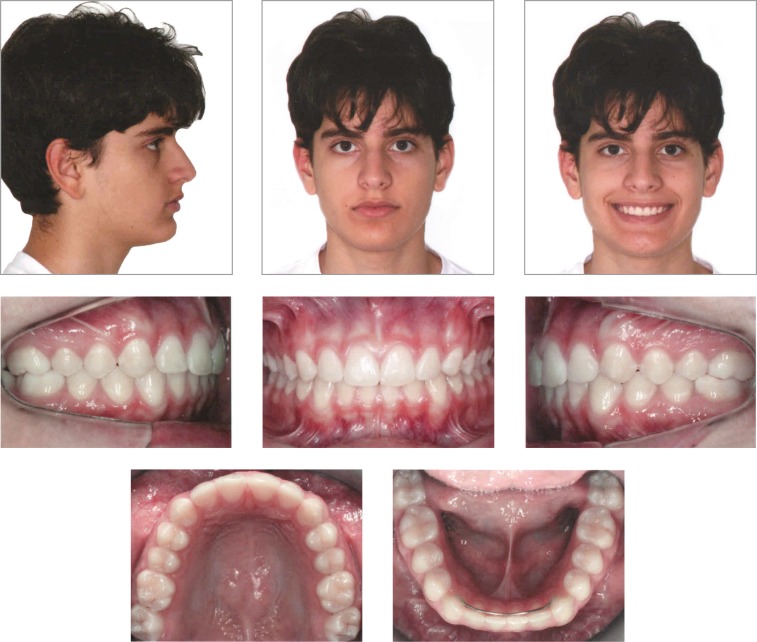
Final facial and intraoral photographs.

**Figure 8.**
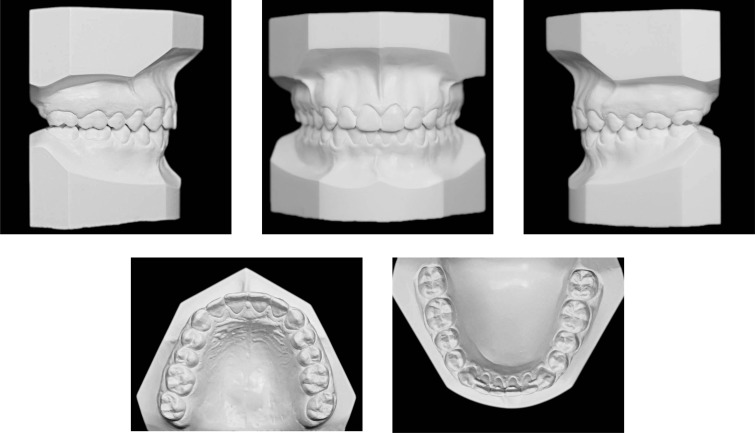
Final casts.

**Figure 9.**
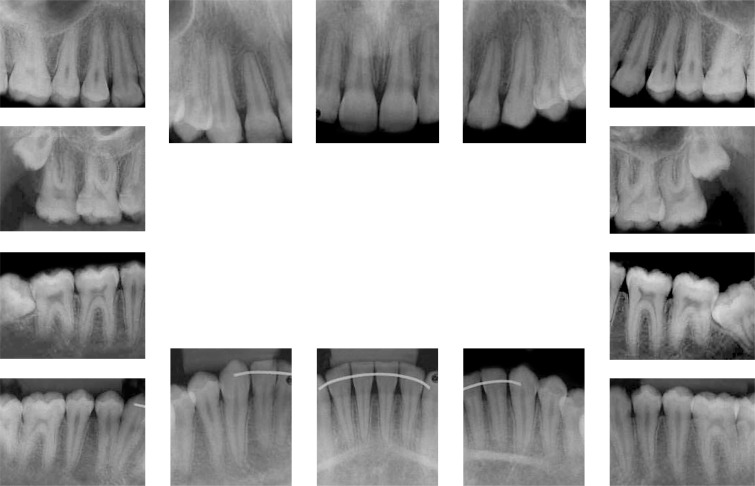
Final periapical radiographs.

**Figure 10.**
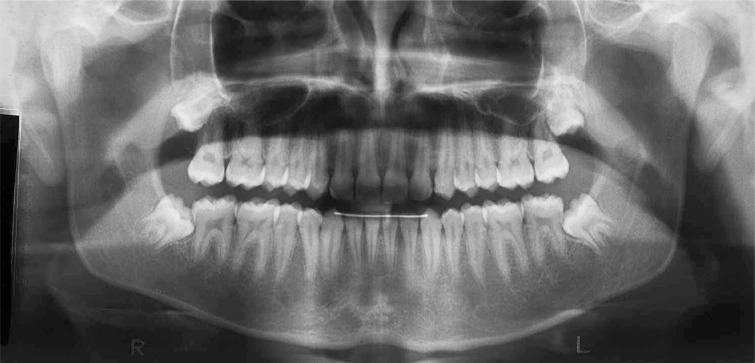
Final panoramic radiograph.

**Figure 11.**
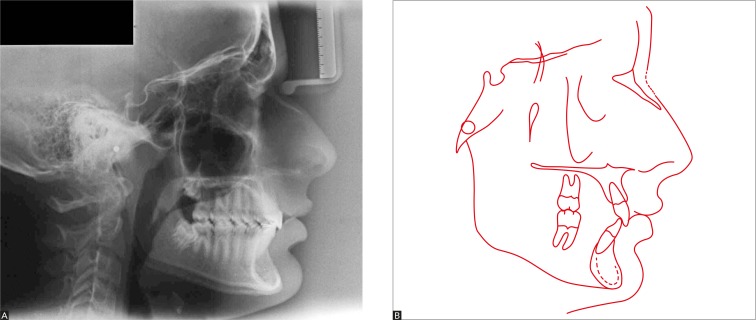
Final lateral cephalogram (A) and cephalometric tracing (B).

**Figure 12.**
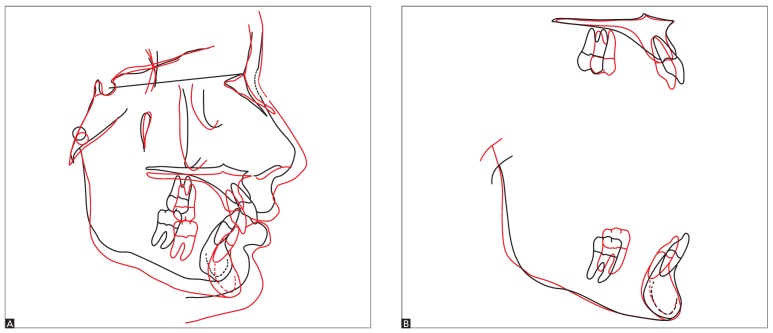
Total (A) and partial (B) initial (black) and final (red) cephalometric tracings superimposition.

Patient's final records (Figs 7-12 and Tab 1) assessment revealed that treatment objectives were achieved. Figure 7 shows that despite mild concave facial profile due to accentuated growth of the chin and nose, there was significant improvement in the relationship established between upper and lower lips, which resulted in passive lip sealing and, as a result, improved facial esthetics. Smile was more harmonious with reduction in buccal corridor width, thereby presenting satisfactory maxillary incisors exposure.

Dental assessment (Figs 7 and 8) revealed significant improvement in the shape of the maxillary arch due to correction of maxillary atresia. Alignment and leveling were successfully achieved. Class I molar and canine relationship was achieved on both sides. Overbite and overjet were corrected.

There was an increase in maxillary (from 51.5 mm to 58 mm) and mandibular intermolar width (from 47.5 mm to 51 mm). Since permanent mandibular incisors had not yet erupted at treatment onset, mandibular intercanine width could not be assessed. Nevertheless, maxillary intercanine width increased from 33.5 mm to 38 mm. Treatment finishing achieved balanced occlusion, with functional harmony in protrusive excursion as well as right and left lateral excursion. Importantly, final results were achieved by means of mild apical remodeling of maxillary incisors despite significant repositioning of these teeth (Fig 9). Moreover, as shown by final panoramic radiograph (Fig 10), good root parallelism was attained in both maxillary and mandibular arches.

As expected, several skeletal changes were achieved (Fig 11 and Tab 1), with significant improvements in sagittal relationship between maxilla and mandible (SNA = 81^o^, SNB = 80^o^ and ANB = 1^o)^. The headgear appliance allowed not only the direction of maxillary growth to change, but also the expression of potential mandibular growth, even though orthopedic effects were produced by the application of mild forces (300 g/side).^2^ There was remarkable reduction in facial convexity and, despite cervical pull, opening of the mandibular plane angle did not occur (GoGn-SN and FMA remained practically unaltered). Significant alteration was also found in the position of maxillary incisors (1-NA = 25^o^ and 5 mm), which notably contributed to overjet correction and improvement of interincisal angle (1 / 1 = 126^o)^.

Total cephalometric superimposition (Fig 12) revealed restricted anterior maxillary growth with downward displacement, only. Nevertheless, there was mild anterior displacement of the mandible, as well, which was responsible for patient's mild concave profile. This fact was greatly reinforced by significant anterior growth of the chin. Overjet and overbite were corrected. Partial maxillary superimposition revealed expressive lingual movement of incisors, with altered tipping and posterior displacement of A Point. A large amount of vertical growth was observed in the mandible, followed by compensatory alveolar growth in the region of molars.

## FINAL CONSIDERATIONS

Angle Class II malocclusion is occasionally associated with a narrow maxilla, which most of times creates the need to start orthodontic treatment by correcting maxillary transverse deficiency for subsequent correction of sagittal relationship.^3^ In the case reported herein, the patient was at a fairly favorable age; for this reason, rapid maxillary expansion by means of Haas appliance was the technique of choice.^4^ After maxillary expansion, an increase in maxillary arch width and spontaneous gain in the mandibular arch were achieved - probably due to alterations in muscle balance between the tongue and buccinator muscles (which affect the increase in mandibular arch width) - with an increase of 3.5 mm in intermolar width.^5^^,^ 6

Overbite correction was achieved by means of leveling the mandibular arch, which had accentuated curve of Spee, by means of intrusion of mandibular incisors. In 1938, Hemley^7^ described treatment carried out by means of anterior bite plate used to favor extrusion of posterior teeth, after which a cervical headgear associated with bite plate was used, yielding satisfactory clinical outcomes when treating Angle Class II malocclusion patients.

As previously reported, even though an acrylic anterior bite plate was used in association with cervical headgear (Kloehn),^8^ patient's mandibular plane remained practically unaltered, probably due to the prevalence of horizontal growth pattern. Combined with accentuated growth of the chin and nose, this fact contributed to render patient's profile slightly concave.^9^^,^ 10

Thus, reassessment of patient's final records confirms that treatment objectives were successfully achieved with Class I molar and canine relationship.^11^ Moreover, nasal breathing was reestablished, thereby eliminating the need for speech therapy. It is worth noting that, despite patient's and his family's opposition, maxillary and mandibular third molars were eventually extracted.
